# The Late Sir Astley Cooper

**Published:** 1844-11-02

**Authors:** 


### THE LATE SIR ASTLEY COOPER.

A statue has but now been placed in St. Paul's Cathedral to the memory of Sir Astley Cooper, the eminent surgeon. It was raised by a public subscription, confined to the profession of which he was so valued a member. The greater portion of the donors were pupils of the late Sir Astley Cooper, headed by Mr. Callaway and Mr. Travers. The staute, exclusive of the pedestal, is eight feet high, and the likeness is considered good. It is by Mr. Baily, the royal academician.—*London Lancet.*

